# Body surface area-based kidney length percentiles misdiagnose small kidneys in children with overweight/obesity

**DOI:** 10.1007/s00467-022-05718-8

**Published:** 2022-09-02

**Authors:** Pierluigi Marzuillo, Gemma Carreras-Badosa, José-María Martínez-Calcerrada, Stefano Guarino, Pier Luigi Palma, Delfina Petrone, Emanuele Miraglia del Giudice, Judit Bassols, Abel López-Bermejo

**Affiliations:** 1 Department of Woman, Child and of General and Specialized Surgery, Università Degli Studi Della Campania “Luigi Vanvitelli”, Via Luigi De Crecchio 2, 80138 Naples, Italy; 2 Pediatric Endocrinology Research Group, Girona Institute for Biomedical Research (IDIBGI), 17190 Salt, Spain; 3 Maternal-Fetal Metabolic Research Group, Girona Institute for Biomedical Research (IDIBGI), 17190 Salt, Spain; 4 Pediatric Endocrinology, Dr. Josep Girona Hospital, 17007 Girona, Spain; 5 Department of Medical Sciences, University of Girona, 17003 Girona, Spain

**Keywords:** Kidney length, Obesity, Overweight, Body surface area, Percentiles, Kidney hypoplasia

## Abstract

**Background:**

We evaluated the diagnostic performance of height-, age- and body surface area (BSA)-based kidney length (KL) percentiles in the identification of at least one small kidney (KL < 3^rd^) and in the prediction of reduced estimated glomerular filtration rate (eGFR) and/or elevated blood pressure (BP) in children with and without overweight (OW)/obesity(OB).

**Methods:**

In this cross-sectional study, 744 apparently healthy children (mean age 8.3 years) were recruited in a primary care setting. Clinical data were collected, and serum creatinine and KL were measured. Height-, age- and BSA-based percentiles of KL were calculated and the association of at least one small kidney per subject with reduced eGFR and/or elevated BP was explored by logistic regression.

**Results:**

Two hundred fifty-seven out of seven hundred forty-four (34.5%) subjects were OW/OB and 127 (17.1%) had reduced eGFR or elevated BP. In separate analyses in children with OW/OB, the KL percentiles calculated on the basis of BSA were lower compared with height- and age-based KL percentiles. Consequently, the prevalence of a small kidney was significantly higher when evaluating percentiles of KL based on BSA compared with other percentiles. In logistic regression analysis, a small kidney was significantly associated with reduced eGFR and/or elevated BP only when using height-based KL percentiles. The KL percentiles according to BSA for the ideal weight (iBSA) showed similar performance compared with height-based percentiles. No differences in the diagnostic performance of different percentiles were found in children with normal weight.

**Conclusions:**

BSA-based percentiles underestimate KL in children with OW/OB. In these subjects, the use of height-based or iBSA-based percentiles should be preferred.

**Graphical abstract:**

**Figure:**
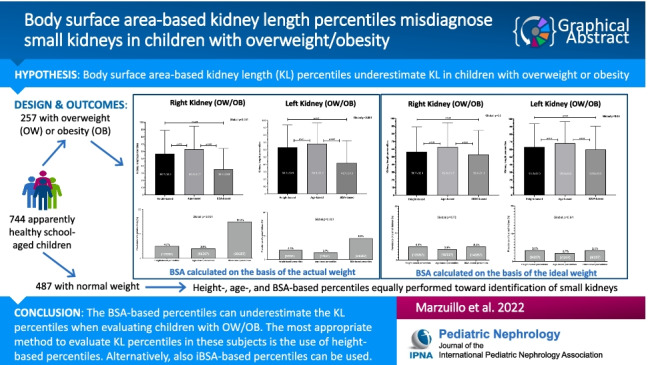
A higher resolution version of the Graphical abstract is available as Supplementary information

**Supplementary Information:**

The online version contains supplementary material available at 10.1007/s00467-022-05718-8.

## Introduction

It is common practice to normalize kidney length (KL) by body surface area (BSA), based on the Mosteller [1] or Du Bois and Du Bois [2] formulae, using height and weight measurements. However, the kidneys only clear the extracellular water and not the fat mass.

It has been suggested to use ideal body weight to calculate BSA, rather than actual body weight [3], but this is rarely practiced. Since in children and adolescents with overweight (OW) or obesity (OB) the actual weight would lead to a higher BSA than BSA using ideal body weight, we hypothesized that KL percentiles would be underestimated in this group of patients despite the fact that they represent a peculiar category of subjects in whom the size of abdominal organs may increase consistently with their BMI [4–6]. Children with OB, in fact, have longer kidneys than their normal weight (NW) counterparts [5]. Normal limits of KL based on height for this group to avoid unnecessary evaluation for nephromegaly have thus been calculated [5].

In view of the OW/OB rates among children and adolescents worldwide with about one-third of children presenting with OW or OB [7, 8], it is important to know the degree of misdiagnosing kidney hypoplasia when the actual weight to calculate BSA is used, because this could lead to unnecessary investigations to rule out chronic kidney disease [9, 10]. We, therefore, conducted a study to identify the most appropriate method to calculate KL percentiles in children with OW/OB. To achieve this aim, we evaluated the diagnostic performance of height-, age- and BSA-based KL percentiles [11] in the identification of at least one small kidney (KL < 3^rd^) and in the prediction of reduced estimated glomerular filtration rate (eGFR; < 90 mL/min/1.73 m^2^) and/or elevated BP (> 95^th^ percentile for age and sex). We also estimated the economic and biological costs derived from a potential small kidney misdiagnosis in children and adolescents with OW/OB.

## Methods

In this study, 744 apparently healthy school-age children (coming from the general population and without a prior diagnosis of disease) were seen in a primary care setting (primary care centers of Girona and Figueres, both regions in Northeastern Spain) and recruited between 2009 and 2015 in a prospective longitudinal study of obesity and cardiovascular risk factors in children [12, 13].

Children were invited to participate in the study during their routine healthy visits in the primary care centers. These visits are part of the protocol of the Childhood Health Program established by the Catalan Public Health Agency. During the visit, the pediatrician explained the study to the families, and those interested to participate were contacted by the study investigators. Informed written consent was signed by the parents before the enrollment.

Inclusion criteria were the availability of ultrasound of both kidneys and the anthropometrical parameters allowing the calculation of height-, age-, and BSA-based KL percentiles [11].

In case of major known congenital anomalies (abnormal liver, kidney, or thyroid functions), evidence of chronic or acute illness, or prolonged use of medication, the patients were excluded from the study. The study was approved by the Institutional Review Board of Dr. Josep Trueta Hospital and was carried out according to the Declaration of Helsinki.

### Assessment of subjects

Subjects were weighed on a calibrated scale and their height was measured with a Harpenden stadiometer with an accuracy of 0.1 cm and 0.05 kg, respectively.

Body-mass index (BMI) was calculated as weight (in kilograms) divided by the square of height (in meters). Age- and sex-adjusted percentiles for BMI were calculated using regional normative data [14]. The study subjects were grouped according to their BMI into normal weight (NW) if BMI was less than 85^th^ percentile, OW if BMI between 85^th^ to less than 95^th^ percentile and OB if BMI was 95^th^ percentile or greater [15]. BSA was calculated as follows: BSA (m^2^) = square root of [height (cm) × weight (kg)/3600] [1]. Also, ideal BSA (iBSA) was calculated computing into the above-mentioned equation the ideal weight of the subject as follows: iBSA (m^2^) = square root of [height (cm) × ideal weight (kg)/3600]. The ideal body weight was the weight at the same percentile as the height, for the same age and gender [16].

Systolic and diastolic blood pressures (SBP and DBP) were measured using an electronic sphygmomanometer (Dinamap Pro 100, GE Healthcare, Chalfont St. Giles, UK) after a 10 min rest on the right arm for three consecutive times with the child in the supine position. The average of the two most similar measurements was used in the analysis. Elevated BP was defined by SBP or DBP > 95^th^ percentile for age, height, and gender [17] for this dataset. KL was measured by high-resolution ultrasonography (MyLabTM25, Esaote, Firenze, Italy) as previously reported [13]. The studies were conducted by an experienced pediatric technician using a 3.5–5 MHz convex transducer. KL was measured as the distance between the upper and the lower pole for each kidney on images taken longitudinally. The measures were taken with the child placed in the supine position and the technician situated on the right side of the child. Thereafter, all KL measurements were taken from the right and left flank of the child. Averages of three measurements were used in the study. The intra-observer error of KL measurement was 2.5% and the inter-observer error was 0%, as all ultrasound measurements were performed by the same observer who was unaware of the clinical and laboratory characteristics of the subjects. Intra-subject coefficient of variation for ultrasound measurements was less than 6%. The percentage of coefficient of variation for each sample was calculated in a smaller subset of children (n = 10) as the standard deviation of the 3 independent measurements, divided by the mean and multiplied by 100.

Height-, age- and BSA-based percentiles of both kidneys on the basis of the normative values provided by Obrycki et al. were calculated [11].

Blood samples were obtained in the morning after an overnight fast. Serum creatinine concentrations were routinely assessed in the clinical laboratory of the Hospital using the enzymatic method (COBAS 702, Roche Diagnostics, IN, USA). eGFR was calculated by the pediatric version of the FAS-equation: [eGFR = 107.3/(Scr/Q)] where Q = 0.0270 × Age + 0.232921.

We defined the eGFR as being reduced when it was < 90 mL/min/1.73 m^2^ [18].

The FAS-equation has previously shown the ability to better select children and adolescents with OW/OB with reduced eGFR and worse cardiometabolic profile [19]. This formula, in fact, eliminates a “potential bias” related to the higher stature of children with OW/OB compared with NW subjects matched for age and gender [19].

### Classifications

A kidney with length < 3^rd^ percentile was defined as “small kidney” in this manuscript. Subjects were also classified as having or not OW/OB, reduced eGFR and/or elevated BP as defined above.

### Cost analysis

The direct costs that would have been incurred for a misdiagnosis of small kidney by BSA-based percentiles compared with the height-based percentiles were calculated. We used the reimbursement of the Catalan Health System to estimate these costs as follows: blood sample collection (€9.00), creatinine measurement (€0.93), urinalysis (€2.96), nephrological follow-up visit (€80.00), Tc99m DMSA renal scintigraphy (€53.00), and cystography (€139.00) [20].

To calculate the X-ray dose exposure, we used an estimated mean dose of 0.30 mSv for the Tc99m DMSA renal scintigraphy and an estimated mean dose of 1.85 mSv for cystography [21].

### Statistical analysis

*P* values < 0.05 were considered significant. Differences for continuous variables were analyzed with the independent sample *t*-test for normally distributed variables and with the Mann–Whitney test in case of non-normality. All the data are presented as mean ± standard deviation scores (SDS). Qualitative variables were compared using the chi-squared test.

Univariate and multivariate logistic regression models were used to explore associations with reduced eGFR and/or elevated BP of a small kidney according to height-, age-, and BSA-based percentiles [11]. We computed into the multivariate logistic regression analyses the parameters showing significant differences (*p* < 0.05) in univariate analysis.

The Stat-Graph XVII software for Windows was used for all statistical analyses except for logistic regression models, which were carried out with SPSS 25 software for Windows.

## Results

### General characteristics

Participation in the study was 70% out of all invited families. We enrolled 744 children, 395 (53.1% boys), with a mean age of 8.3 years (age range: 3.2–14.8 years). The general characteristics of the enrolled subjects are shown in Table 1. Out of these 744 subjects, 257 (34.5%) were OW/OB and 127 (17.1%) had reduced eGFR or elevated BP. Specifically, four children showed both eGFR reduction and elevated BP, 77 only eGFR reduction and 46 only elevated BP. On the basis of weight status, 41 out of 257 (15.9%) children with OW/OB showed eGFR reduction and/or elevated BP compared with 86 out of 487 (17.6%) children with normal weight (NW) (*p* = 0.55). Children with OW/OB were older compared with those with NW (9.0 ± 2.0 years vs. 7.9 ± 1.9 years; *p* < 0.001).

**Table 1:** **General characteristics of the enrolled patients.** Continuous variables are presented as median and interquartile range, if not normally distributed, and as mean and standard deviation, if normally distributed

Age (years), mean (SD)	8.3 (2.0)
Gender (female), No. (%)	349 (46.9)
Puberty (yes), No. (%)	99 (13.3)
BMI-SDS, median (IQR)	0.4 (2.3)
Height-SDS, median (IQR)	0.5 (1.6)
BSA (m^2^), median (IQR)	1.1 (0.4)
Waist/height ratio	0.5 (0.1)
SBP-SDS, mean (SD)	0.6 (0.9)
DBP-SDS, median (IQR)	0.1 (0.9)
Creatinine (mg/dL), median (IQR)	0.5 (0.12)
eGFR (mL/min/1.73 m^2)^, median (IQR)	99.4 (23.4)

*BMI*, body mass index; *BSA*, body surface area; *DBP*, diastolic blood pressure; *eGFR*, estimated glomerular filtration rate; *IQR*, interquartile range; *SBP*, systolic blood pressure; *SD*, standard deviation; *SDS*, standard deviation scores

### Comparison of performance of height-, age- and BSA-based KL percentiles

**Fig. 1:**
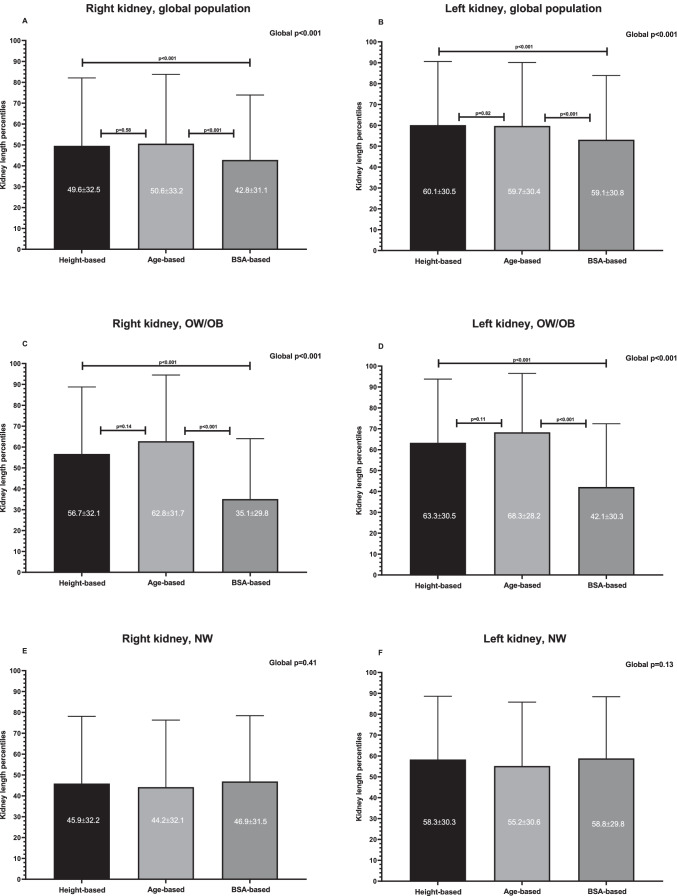
**Comparison of the kidney length percentiles calculated on the basis of height, age and BSA in all children and in those with overweight/obesity or with normal weight.** *Abbreviations:* BSA, body surface area; OB, obesity; OW, overweight; NW, normal weight

**Fig. 2:**
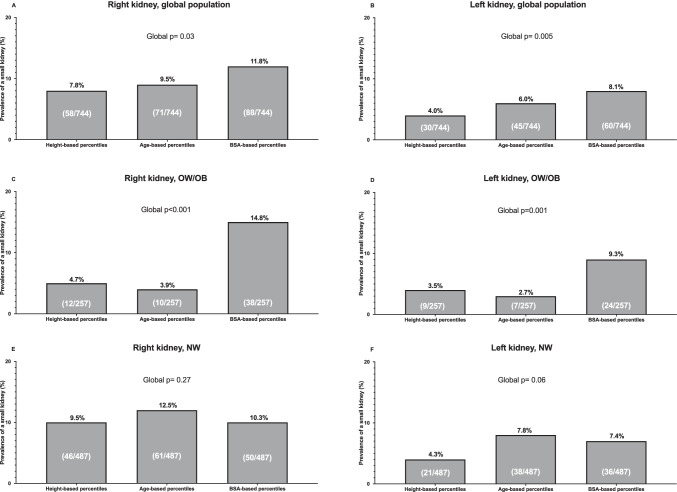
**Prevalence of a small kidney comparing height-, age-, and BSA-based percentiles in all children and in those with overweight/obesity or with normal weight.** *Abbreviations:* BSA, body surface area; OB, obesity; OW, overweight; NW, normal weight

When evaluating the global population (including subjects with NW, OW and OB), the KL percentiles calculated on the basis of BSA were significantly lower compared with height- and age-based percentiles. The results were similar for both kidneys (Figs. 1A and B). Moreover, the prevalence of a small kidney was significantly higher when evaluating KL by BSA-based percentiles compared with height- and age-based percentiles (Figs. 2A and B).

Restricting the analysis to the children with OW/OB, for both kidneys, the KL percentiles calculated on the basis of BSA were lower compared with height- and age-based KL percentiles (Figs. 1C and D), and therefore the prevalence of a small kidney was significantly higher when evaluating KL by BSA-based percentiles compared with height- and age-based percentiles (Figs. 2C and D).

When the analysis was limited to the children with NW, the KL percentiles calculated on the basis of height, age and BSA (Figs. 1E and F) and the prevalence of a small kidney on the basis of height, age and BSA percentiles (Figs. 2E and F) were similar for both kidneys.

An exploratory analysis of the prediction of reduced eGFR and/or elevated BP by a small kidney on the basis of height-, age- and BSA-based KL percentile calculations was performed (Table 2). We found that, for the global population and in univariate analysis, a small kidney on the basis of height, age and BSA percentiles was significantly associated with reduced eGFR and/or elevated BP (Table 2). In multivariate analysis, only a small kidney on the basis of height persisted significantly associated with reduced eGFR and/or elevated BP (Table 2). Assessing separately children with OW/OB, a small kidney was significantly associated with reduced eGFR and/or elevated BP only when calculated on the basis of height (Table 2). On the other hand, among subjects with NW, in univariate analysis, a small kidney calculated on the basis of height-, age-, or BSA-derived KL percentiles was significantly associated with reduced eGFR and/or elevated BP (Table 2). None of them persisted as significant in multivariate analysis (Table 2).

**Table 2:** Exploratory analyses of the prediction of reduced eGFR and/or elevated blood pressure by a small kidney (at least one kidney with length < 3th percentile) on the basis of height-, age- and BSA-derived percentiles

	Global population						Children with overweight/obesity						Children with normal weight					
	Univariate analysis			Multivariate analysis			Univariate analysis			Multivariate analysis			Univariate analysis			Multivariate analysis		
KL percentiles	OR	95%CI	*p*	OR	95%CI	*p*	OR	95%CI	*p*	OR	95%CI	*p*	OR	95%CI	*p*	OR	95%CI	*p*
Height-based	5.2	1.6–16.8	0.006	5.0	1.2–20.3	0.02	2.9	1.02–8.3	0.04	2.9	1.02–8.3	0.04	6.2	1.5–26.2	0.01	1.8	0.4–8.3	0.47
Age-based	1.9	1.01–4.0	0.05	1.0	0.4–2.3	0.9	2.2	0.7–7.5	0.19	n.a	n.a	n.a	2.3	1.01–5.2	0.04	1.1	0.4–2.8	0.8
BSA-based	2.1	1.1–4.1	0.03	0.9	0.4–2.1	0.9	1.9	0.8–4.1	0.11	n.a	n.a	n.a	4.4	1.3–14.4	0.01	3.7	0.6–24.0	0.17

### Comparison of performance of height-, age- and iBSA-based KL percentiles in children with OW/OB

**Fig. 3:**
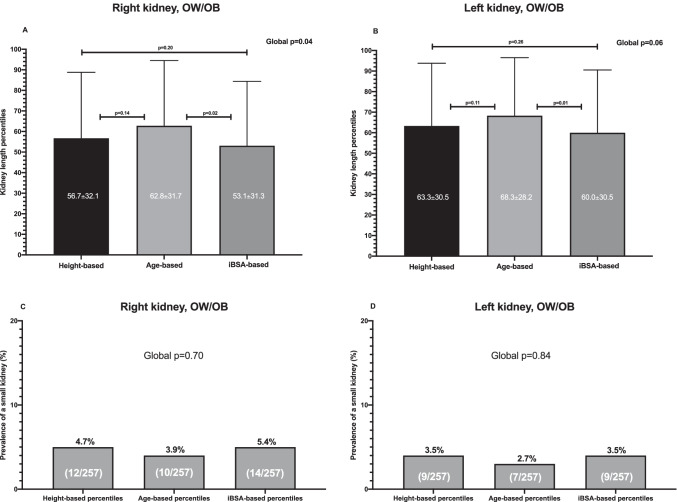
**Comparison of the right and left kidney length percentiles and prevalence of a small kidney on the basis of height, age, and iBSA percentiles in children with overweight/obesity.** *Abbreviations:* iBSA, ideal body surface area; OB, obesity; OW, overweight; NW, normal weight

For both kidneys, the KL percentiles calculated on the basis of iBSA were significantly lower only when compared with age-based percentiles (Figs. 3A and B). The prevalence of a small kidney was similar when evaluating KL by age-based and iBSA-based percentiles (Figs. 3C and D).

In univariate analysis, a small kidney on the basis of both height and iBSA was significantly associated with reduced eGFR and/or elevated BP. None of them persisted as significant in multivariate analysis (Table 3).

**Table 3:** Exploratory analyses of the prediction of reduced eGFR and/or elevated blood pressure by a small kidney (at least one kidney with length < 3^th^ percentile) on the basis of height-, age- and iBSA-derived percentiles in children with OW/OB

	Univariate analysis			Multivariate analysis		
KL percentiles	OR	95%CI	*p*	OR	95%CI	*p*
Height-based	2.9	1.02–8.3	0.04	1.9	0.4–10	0.5
Age-based	2.2	0.7–7.5	0.19	n.a	n.a	n.a
iBSA-based	2.7	1.1–7.3	0.04	1.7	0.3–10	0.6

### Estimate of economic and biological costs derived from a misdiagnosis of a small kidney obtained by BSA-based KL percentiles in children with OW/OB

On the basis of height-based KL percentiles, 18 out of 257 (7%) subjects had a small kidney (three with both small kidneys and 15 with one small kidney). On the basis of age-based KL percentiles 14 out of 247 (5.7%) subjects had a small kidney (three with both small kidneys and 11 with one small kidney). On the basis of BSA-based KL percentiles, 46 out of 257 (17.9%) subjects had a small kidney (16 with both small kidneys and 30 with one small kidney). Because the height-based KL percentiles showed the best diagnostic performance, these percentiles were used as the gold standard in the present analysis. Therefore, compared with height-based percentiles, when using the BSA-based percentiles, 28 out of 257 (10.9%) children received a misdiagnosis of a small kidney with a potential inappropriate indication to undergo further biochemical and instrumental exams.

Considering the performing of blood sample collection with creatinine measurement, urinalysis, nephro-urological visit, Tc99m DMSA renal scintigraphy and cystography for each of these 28 subjects, the cumulative direct economic and biological costs due to small kidney misdiagnosis were respectively 7976.92 € (284.89 € for each patient) and 81.7 mSv (2.15 mSv for each patient, equivalent to 107.5 chest X-rays for each patient).

## Discussion

This study investigates the diagnostic performance of BSA-based percentiles of KL compared with height- and age-based percentiles. Our data indicate that the BSA-based percentiles underestimate KL in children and adolescents with OW/OB yielding a misdiagnosis of a small kidney in 11% of these subjects.

A small kidney is associated with reduced eGFR and/or hypertension [9, 22, 23]. This association has been also shown in our population (Table 2). Interestingly, not all percentiles performed similarly in predicting reduced eGFR and/or elevated BP. In the global population, in univariate analysis, a small kidney according to all the percentiles (height-, age- and BSA-based) was significantly associated with reduced eGFR and/or elevated BP. However, in multivariate analysis, only a small kidney calculated on the basis of height-based percentiles showed a significant association with reduced eGFR and/or elevated BP. Similar findings were obtained in children with OW/OB. Moreover, among children with NW, all the percentiles performed equally in the identification of a small kidney. In these subjects, in fact, in univariate analysis, a small kidney calculated on the basis of both height- and age- and BSA-based percentiles was significantly associated with reduced eGFR and/or elevated BP while in multivariate analysis, none of them remained significant.

Usually the right kidney is smaller than the left one [24, 25]; we confirmed this finding also in our pediatric population. In fact, the right KL percentiles both for age, height, BSA and iBSA and both in children with NW and OW/OB were lower compared with the left KL percentile (Fig. 1). Despite this difference, the BSA-based percentiles underestimated the KL of both kidneys compared with other percentiles.

We wish to emphasize that the percentage of subjects showing elevated BP and/or reduced eGFR is quite high (17%) in our study group. This could be explained by the cross-sectional design of the study, with measurement of the parameters only in a 1-day clinical evaluation. In fact, evidence indicates that when repeating the BP measurements in different visits, the percentage of elevated BP significantly decreases from 11.4 to 2.2% [26]. Similarly, one serum creatinine measurement is not sufficient to correctly identify the subjects with reduced eGFR levels because the creatinine measurement is influenced by several factors, such as dietary intake, body composition and muscle mass [27]. Moreover, as stated in the KDIGO chronic kidney disease guidelines, the serum creatinine measurement over a 3-month period is mandatory to correctly identify patients with chronic kidney disease [10].

The presence of a small kidney predicted the presence of reduced eGFR and/or elevated BP in our study group despite the overestimation of the subjects with these conditions. This adds further evidence to the association between small kidney and reduced eGFR and/or elevated BP and reinforces the importance of a correct interpretation of KL using the most appropriate percentiles according to weight status.

Our data indicate that when evaluating children with OW/OB, the most appropriate percentiles are those calculated on the basis of height. This finding is in line with the results of Obrycki et al. [11] who showed — with different methods and starting from a different research question — that the most significant predictor of KL was statural height. Differently from the above-mentioned paper [11], we tested the performance of the different percentiles [11] toward the diagnosis of small kidney after the classification of population in NW and OW/OB. In addition, we showed that in children with OW/OB, as an alternative, also the BSA-based percentiles can be used but it is important to calculate the subject’s BSA on the basis of ideal weight (iBSA). In this manner, a similar performance compared with height-based percentiles could be obtained in the identification of a small kidney. In fact, differences among the percentiles of KL on the basis of iBSA, height, and age were minimal (Fig. 3) and the prevalence of a small kidney was similar when using height- and iBSA-based KL percentiles (Fig. 3). Moreover, height- and iBSA-based KL percentiles performed equally well in the identification of reduced eGFR and/or elevated blood pressure by a small kidney (Table 3).

Correct identification of a small kidney is also important because it can reflect congenital anomalies of the kidney and urinary tract. The current clinical practice relies on performing a Tc99m DMSA renal scintigraphy and cystography in the case of a small kidney [9, 28].

Our cost analysis in case of misdiagnosis of a small kidney using BSA-based percentile to evaluate KL in children with OW/OB indicates both non-negligible economic and biological costs further underlining the importance of a correct selection of KL percentiles to be used in clinical practice in case of children with OW/OB. Moreover, the reduction of X-ray exposure is mandatory in children because evidence exists linking X-ray exposure, especially if occurring early in life, and increased risk of cancer during the life of the subjects [29].

Finally, we wish to emphasize that the prevalence of 34.5% of OW/OB found among Spanish children and adolescents in our study is in line with published data about children and adolescents of the same nation indicating a prevalence of OW/OB of 35.3% [30].

Limitations of our study include the cross-sectional design with the lack of a second collection of parameters within the following 3 months in subjects with elevated BP and/or reduced eGFR and the lack of availability of data regarding proteinuria.

Unfortunately, low agreement between kidney volume and KL has been demonstrated [31–33]. As a future perspective, it could be interesting to study the performance of kidney volume to precisely assess kidney size in children and adolescents with OW/OB. In the meantime, when evaluating KL in children with OW/OB, the KL percentiles could be evaluated on the basis of height or iBSA.

In conclusion, our study shows that the BSA-based percentiles can underestimate the KL percentiles when evaluating children with OW/OB. The most appropriate method to evaluate KL percentiles in these subjects is the use of height-based percentiles. Alternatively, also iBSA-based percentiles — by calculating BSA on the basis of ideal weight — can be used with a similar diagnostic performance in the detection of a small kidney and in the identification of subjects with elevated BP and/or reduced eGFR.

## Supplementary Information

Below is the link to the electronic supplementary material.

Graphical Abstract (PPTX 3525 KB)

## Data Availability

The dataset generated during and/or analysed during the current study are available from the corresponding author on reasonable request.
