# Supplementary material for: Body surface area-based kidney length percentiles misdiagnose small kidneys in children with overweight/obesity
Source: Pediatr Nephrol. 2022 Sep 2;38(5):1523–32. doi: 10.1007/s00467-022-05718-8 (PMC10060296; doi:10.1007/s00467-022-05718-8)
Supplement: Supplementary file 1 — Graphical Abstract (PPTX 3525 KB) [file 467_2022_5718_MOESM1_ESM.pptx]

## Slide 1
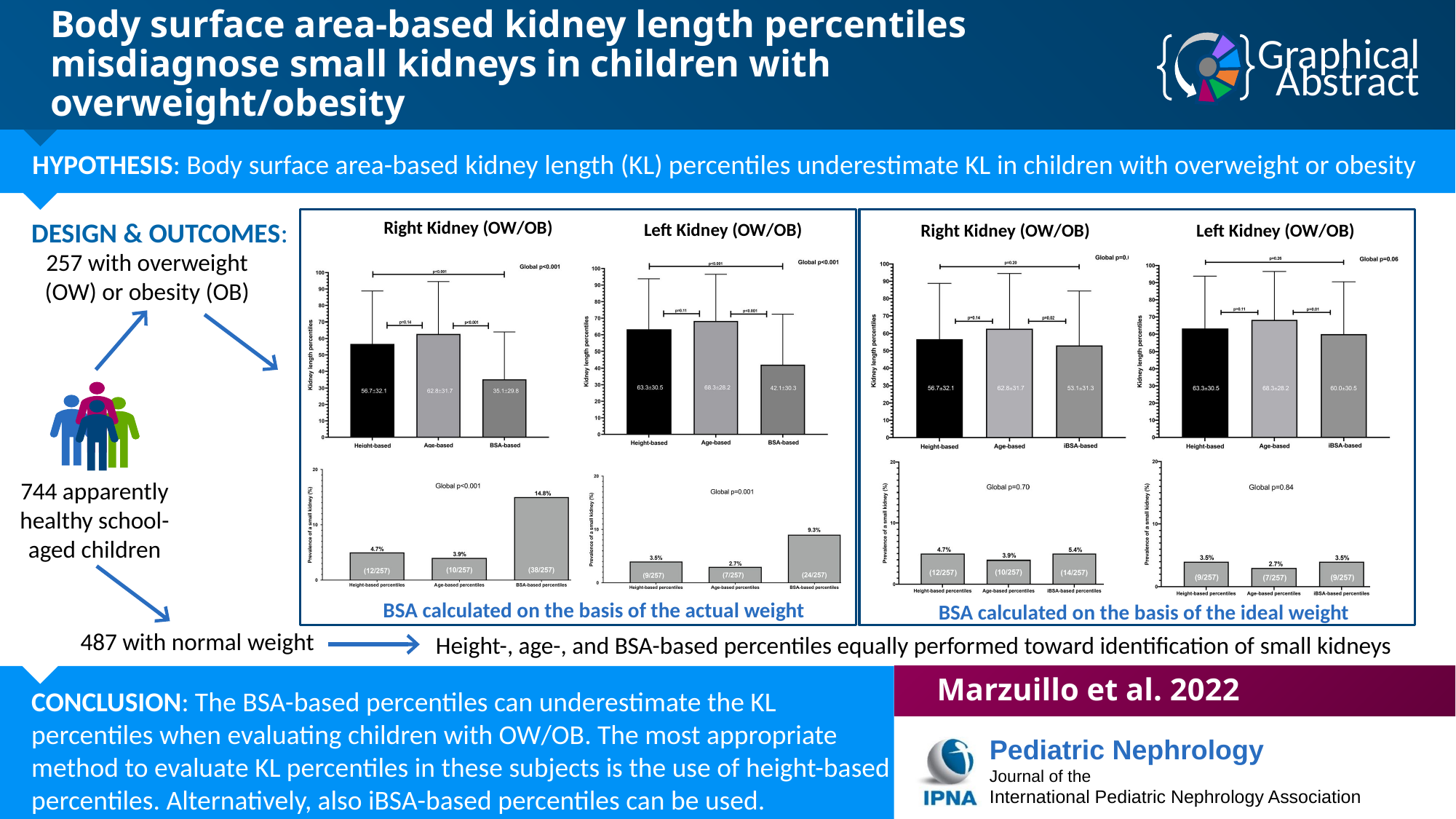

Body surface area-based kidney length percentiles misdiagnose small kidneys in children with overweight/obesity
HYPOTHESIS: Body surface area-based kidney length (KL) percentiles underestimate KL in children with overweight or obesity
Right Kidney (OW/OB)
Left Kidney (OW/OB)
Left Kidney (OW/OB)
Right Kidney (OW/OB)
BSA calculated on the basis of the actual weight
BSA calculated on the basis of the ideal weight
DESIGN & OUTCOMES:
257 with overweight (OW) or obesity (OB)
744 apparently healthy school-aged children
487 with normal weight
Height-, age-, and BSA-based percentiles equally performed toward identification of small kidneys
Marzuillo et al. 2022
CONCLUSION: The BSA-based percentiles can underestimate the KL percentiles when evaluating children with OW/OB. The most appropriate method to evaluate KL percentiles in these subjects is the use of height-based percentiles. Alternatively, also iBSA-based percentiles can be used.
